# An Equation Based on Fuzzy Mathematics to Assess the Timing of Haemodialysis Initiation

**DOI:** 10.1038/s41598-018-37762-6

**Published:** 2019-04-10

**Authors:** Ying Liu, Degang Wang, Xiangmei Chen, Xuefeng Sun, Wenyan Song, Hongli Jiang, Wei Shi, Wenhu Liu, Ping Fu, Xiaoqiang Ding, Ming Chang, Xueqing Yu, Ning Cao, Menghua Chen, Zhaohui Ni, Jing Cheng, Shiren Sun, Huimin Wang, Yunyan Wang, Bihu Gao, Jianqin Wang, Lirong Hao, Suhua Li, Qiang He, Hongmei Liu, Fengmin Shao, Wei Li, Yang Wang, Lynda Szczech, Qiuxia Lv, Xianfeng Han, Luping Wang, Ming Fang, Zach Odeh, Ximing Sun, Hongli Lin

**Affiliations:** 1 Dalian Medical University Graduate School, Dalian, China; 2 Department of Nephrology, The First Affiliated Hospital of Dalian Medical University, Liaoning Province Translational Medicine Research Center of Kidney Disease, Dalian, China; 3 Kidney Research Institute of Dalian Medical University, Dalian, China; 4 School of Control Science and Engineering, Dalian University of Technology, Dalian, China; 5 Department of Nephrology, Chinese PLA General Hospital, Chinese PLA Institute of Nephrology, State Key Laboratory of Kidney Diseases, National Clinical Research Center for Kidney Diseases, Beijing Key Laboratory of Kidney Disease Research, Beijing, China; 6 School of Economics, Dongbei University of Finance and Economics, Dalian, China; 7 Blood Purification Center, The First Affiliated Hospital of Xi’an Jiaotong University, Xi’an, China; 8 Division of Nephrology, Guangdong General Hospital, Guangdong Academy of Medical Sciences, Guangzhou, China; 9 Division of Nephrology, Beijing Friendship Hospital, Capital Medical University, Beijing, China; 10 Kidney Research Institute, Division of Nephrology, West China Hospital of Sichuan University, Chengdu, China; 11 Division of Nephrology, Zhongshan Hospital, Fudan University, Shanghai, China; 12 Division of Nephrology, Dalian Municipal Central Hospital, Dalian, China; 13 Department of Nephrology, The First Affiliated Hospital, Sun Yat-sen University, Key Laboratory of Nephrology, Ministry of Health of China, Guangzhou, China; 14 Blood Purification Center, General Hospital of Shenyang Military Area Command, Shenyang, China; 15 Department of Nephrology, General Hospital of Ningxia Medical University, Yinchuan, China; 16 Department of Nephrology, Renji Hospital, School of Medicine, Shanghai Jiaotong University, Shanghai, China; 17 Division of Nephrology, Huashan Hospital, Fudan University, Shanghai, China; 18 Department of Nephrology, Xijing Hospital, the Fourth Military Medical University, Xi’an, China; 19 Division of Nephrology, General Hospital of Benxi Iron and Steel Co., Ltd, Benxi, China; 20 Blood Purification Center, Daping Hospital & Surgery Institute, the Third Military Medical University, Chongqing, China; 21 Division of Nephrology, Affiliated Zhongshan Hospital of Dalian University, Dalian, China; 22 Division of Nephrology, Lanzhou University Second Hospital, Lanzhou, China; 23 Division of Nephrology, the First Affiliated Hospital of Harbin Medical University, Harbin, China; 24 Division of Nephrology, the First Affiliated Hospital of Xinjiang Medical University, Urumchi, China; 25 Division of Nephrology, Zhejiang Provincial People’s Hospital, Hangzhou, China; 26 Division of Nephrology, An Steel Group Hospital, Anshan, China; 27 Blood Purification Center, The People’s Hospital of Zhengzhou University & Henan Provincial People’s Hospital, Zhengzhou, China; 28 Medical Research & Biometrics Center, Fuwai Hospital, Chinese Academy of Medical Sciences, Beijing, China; 29 FibroGen, Inc, San Francisco, CA USA

## Abstract

In order to develop an equation that integrates multiple clinical factors including signs and symptoms associated with uraemia to assess the initiation of dialysis, we conducted a retrospective cohort study including 25 haemodialysis centres in Mainland China. Patients with ESRD (n = 1281) who commenced haemodialysis from 2008 to 2011 were enrolled in the development cohort, whereas 504 patients who began haemodialysis between 2012 and 2013 were enrolled in the validation cohort comprised. An artificial neural network model was used to select variables, and a fuzzy neural network model was then constructed using factors affecting haemodialysis initiation as input variables and 3-year survival as the output variable. A logistic model was set up using the same variables. The equation’s performance was compared with that of the logistic model and conventional eGFR-based assessment. The area under the bootstrap-corrected receiver-operating characteristic curve of the equation was 0.70, and that of two conventional eGFR-based assessments were 0.57 and 0.54. In conclusion, the new equation based on Fuzzy mathematics, covering laboratory and clinical variables, is more suitable for assessing the timing of dialysis initiation in a Chinese ESRD population than eGFR, and may be a helpful tool to quantitatively evaluate the initiation of haemodialysis.

## Introduction

Maintenance haemodialysis is the main renal replacement therapy used for patients with end-stage renal disease (ESRD). However, the optimal time of haemodialysis initiation remains a vital factor to reduce the morbidity of complications and mortality associated with dialysis^1–4^. The early initiation of haemodialysis leads to an accelerated decline in residual renal function, poor quality of life, and waste of medical resources. In contrast, the late initiation of haemodialysis increases the incidence of complications, causes higher mortality, and increases treatment costs. Over the past three decades, several studies have attempted to assess the initiation time of dialysis; however, no consensus has been reached.

From the 1970s to 1990s, studies have shown that a high initial KT/V level (K, dialyzer clearance of urea; t, dialysis time; V, volume of the distribution of urea) for urea can improve dialysis outcomes^5–7^. In the USA, the mean estimated GFR (eGFR) at dialysis initiation has gradually increased from 1996 until 2008. In particular, the proportion of patients beginning haemodialysis with an eGFR of >10 mL/min has increased from 20% to 52%, whereas those beginning haemodialysis with an eGFR of ≥15 mL/min has increased from 4% to 17%^8^. However, subsequent observational studies based on these registries included a large number of patients, but produced controversial results^9–14^. In particular, the IDEAL study in 2010 showed that survival did not significantly differ between ESRD patients with early and late dialysis initiation^14^.

One reason for the conflicting results was that the definitions of “early” and “late” were based on serum creatinine-based GFR estimations, including equations such as the Modification of Diet in Renal Disease (MDRD) study equation^15^. The equations for GFR estimation do not consider essential clinical factors such as nutrition, diabetes mellitus, and signs and symptoms of uraemia (e.g., volume overload, gastrointestinal tract symptoms, and anaemia), which may affect dialysis initiation. Therefore the updated 2015 Kidney Disease Outcomes Quality Initiative (KDOQI) guidelines recommend that the decision to initiate dialysis should be based on an assessment of multiple factors, such as signs and symptoms of, for example, uraemia, volume overload, and heart failure, and not only the eGFR level^16^. However, these clinical factors are subjectively assessed by doctors and depend on their individual experience. Hence, the quantitative assessment of these aforementioned clinical factors may enhance the accuracy of the assessment of the timing of haemodialysis initiation, especially for doctors who lack extensive clinical experience.

However, it is difficult to quantify these ‘fuzzy’ clinical factors, especially the non-linear relationships between these factors and outcomes, using traditional statistical methods^17^. In the present study, we adopted ‘fuzzy’ methods instead of traditional statistical methods. In past decades, the development of fuzzy mathematics has impacted the fields of modelling because it can describe vague statements^18^. Artificial neural networks (ANNs), as one of the widely used techniques of fuzzy mathematics, have the advantage of being able to detect complex, non-linear problems^19^. Generally, ANNs consist of multiple layers; hence the information transfers from the input layer to the output layer of the neuronal network layer by layer. They can be calibrated using almost any type of input data (i.e., assumed risk factors), and the output can be one-dimensional or high dimensional (i.e., outcomes) and can simultaneously consider all possible interactions between those risk factors. Therefore, ANNs have been used to predict technique survival for peritoneal dialysis, and the results showed ANNs have higher accuracy than logistic regression models^20,21^. Marshall *et al*. created an ANN model to predict GFR, which showed better results than algebraic formulas. These studies confirmed that ANNs could deal with dialysis datasets^22^. In our previous study, we established an improved ANN model termed the kernel logistic neural network-restricted Boltzmann machine (KLNN-RBM) to solve complex variable screening problems efficiently^23^.

Moreover, fuzzy neural networks combine the advantages of fuzzy logic in processing vague and uncertain information, and neural networks in good learning abilities. The Takagi–Sugeno (T-S) type fuzzy neural network is the most widely used modelling method among fuzzy neural networks^24^. This technique has been applied in biological and clinical fields for modelling, especially in the processing of time-delay datasets (i.e., risk factors and outcomes) and has produced satisfactory results^25,26^. Therefore, the KLNN-RBM and the T-S type fuzzy neural networks appear to be viable methods to select variables and for modelling for assessing dialysis initiation.

To the best of our knowledge, this is the first report of the use fuzzy mathematics to develop a novel equation to assess dialysis initiation, which we termed as the “dialysis initiation based on fuzzy mathematics equation” (DIFE). Furthermore, we compared the DIFE with the conventional eGFR-based assessment and showed that the DIFE is more accurate to evaluate dialysis initiation. Our results suggest that the DIFE offers a novel method to assess the initiation of dialysis, and may have implications for decision-making related to dialysis initiation in clinical practice.

## Methods

### Study design and participants

This retrospective cohort study encompassed 25 haemodialysis centres covering seven geographical regions of Mainland China. All the study centres serve as quality control centres of blood purification or were recommended by these centres in each province, municipality, or autonomous region.

The protocols were approved by the Ethics Committee of the First Affiliated Hospital of Dalian Medical University; the Chinese PLA General Hospital; the First Affiliated Hospital of Xi’an Jiaotong University; Guangdong General Hospital; Beijing Friendship Hospital; Capital Medical University; West China Hospital of Sichuan University; Zhongshan Hospital; Fudan University; Dalian Municipal Central Hospital; the First Affiliated Hospital; Sun Yat-sen University; the General Hospital of Shenyang Military Area Command; the General Hospital of Ningxia Medical University; Renji Hospital; School of Medicine; Shanghai Jiaotong University; Huashan Hospital; Fudan University; Xijing Hospital; the Fourth Military Medical University; the General Hospital of Benxi Iron and Steel Co., Ltd; Daping Hospital & Surgery Institute; the Third Military Medical University; the Affiliated Zhong Shan Hospital of Dalian University; Lanzhou University Second Hospital; the First Affiliated Hospital of Harbin Medical University; the First Affiliated Hospital of Xinjiang Medical University; Zhejiang Provincial People’s Hospital; An Steel Group Hospital; Henan Provincial People’s Hospital; and The People’s Hospital of Zhengzhou University. We obtained written informed consent from each patient, and personal information was protected during data collection. All the study methods were performed in accordance with relevant guidelines and regulations.

Patients with ESRD who began maintenance haemodialysis between January 1, 2008, and September 30, 2013, were enrolled. Patients were 18–85 years of age, were diagnosed with chronic kidney disease (CKD), had two successive eGFR measurements of ≤30 mL/min/1.73 m^2^ within 3 months before haemodialysis initiation, and had commenced haemodialysis for a minimum of 3 months. The exclusion criteria were as follows: patients who were diagnosed with acute kidney injury (AKI); those who underwent or were scheduled to undergo peritoneal dialysis or kidney transplantation; those with a malignancy that significantly affected survival (e.g., malignant tumours, hepatic cirrhosis); and those who experienced accidental death caused by unexpected reasons, including traffic accidents and suicide.

**Figure 1:**
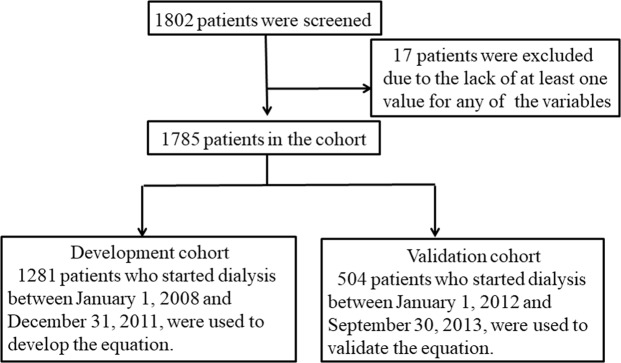
Flowchart of the study.

Of the 1802 patients who began haemodialysis between January 1, 2008, and September 30, 2013, 17 patients with missing serum albumin and serum phosphate data were excluded. The enrolled patients were divided into two cohorts according to the start time of haemodialysis. A total of 1281 patients who started haemodialysis between January 1, 2008, and December 31, 2011, were included as the development cohort, whereas 504 patients who started haemodialysis between January 1, 2012, and September 30, 2013, were retained within the validation cohort (Fig. 1).

### Data collection

Data from the time spanning the initiation of haemodialysis to the outcomes were extracted from the inpatient/outpatient records at the haemodialysis centres by the investigators of each centre. To assess the precision of the determination of clinical signs and symptoms from the medical records, three nephrologists (Y.L., X.H., and L.W.) independently reviewed the data of a random sample of 100 records. The documentation used for abstraction included inpatient medical records for haemodialysis initiation, first-time haemodialysis records, laboratory data, and surgical records for first access. Baseline demographic data and clinical data included sex, birth date, date of haemodialysis initiation, first access, death date, primary disease, and comorbidity. The clinical signs and symptoms at the time of haemodialysis initiation were grouped as follows: heart failure, vomiting, uremic encephalopathy, and oedema grade 2+ and 3+. Determinations of the clinical signs and symptoms were made based on the clinical guidelines by nephrologists during the outpatient or inpatient consultations^16^.

Laboratory data collected within 3 months prior to haemodialysis initiation included haemoglobin, serum albumin, blood urea nitrogen, serum creatinine, serum potassium, serum sodium, free calcium, and serum phosphorus levels. In all the clinical laboratories of the study centres, serum creatinine levels were measured using the sarcosine oxidase method. The eGFR (mL/min/1.73 m^2^) at the initiation of haemodialysis was computed using the Chinese modified MDRD study equation 7: eGFR = 170 × serum creatinine^−0.999^ × age^−0.176^ × blood urea nitrogen^−0.170^ × serum albumin^0.318^ (×0.762 if the patient is female; ×1.202 if the patient is of Chinese descent). All participating investigators were nephrologists and had received uniform study training.

### Outcomes

The primary outcome was all-cause mortality within 3 years after haemodialysis initiation. The dates and reasons of death were obtained from the medical records of the study centres. Survival, expressed as months, was defined as the time from the start date of haemodialysis to the date of death for the patient (within 3 years after initiation) or 36 months for the surviving patients.

### Development of the DIFE

#### Equation variable selection using the KLNN-RBM model

Based on the recommendation of the timing of haemodialysis initiation in the 2015 KDOQI guidelines^16^ and our previous study^27^, 13 candidate variables were considered for inclusion in the DIFE, including age, sex, serum creatinine level, blood urea nitrogen level, serum albumin level, blood haemoglobin level, serum potassium level, serum phosphorus level, heart failure, vomiting, oedema grade 2+ and 3+, uremic encephalopathy, and diabetes mellitus. Sex, diabetes mellitus, heart failure, vomiting, oedema grade 2+ and 3+, and uremic encephalopathy were used as binary variables, and were transformed using dummy variable encoding (e.g., female = 1, male = 0; yes = 1, no = 0).

**Figure 2:**
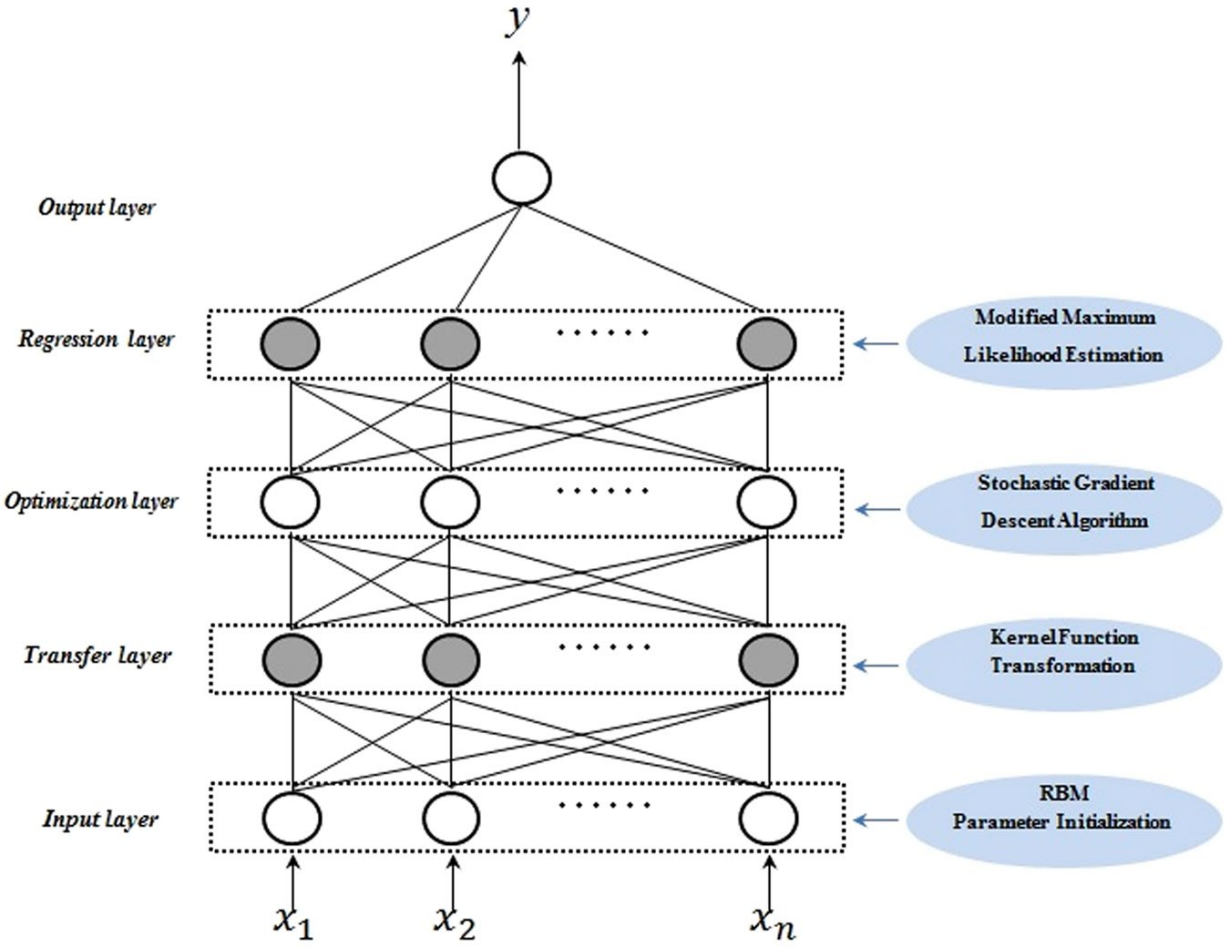
The structure of the kernel logistic neural network-restricted Boltzmann machine (KLNN-RBM) model.

Using the KLNN-RBM model, the variables adopted to establish the DIFE equation were detected in the development cohort. We initialized weight and bias parameters with the help of RBM first, and then optimized the parameters using a modified maximum likelihood estimation and stochastic gradient descent method to obtain higher classification accuracy. The structure of the KLNN-RBM model is shown in Fig. 2. The inputs of the KLNN-RBM model included the candidate variables, whereas the output included the patient survival time after haemodialysis initiation (<12 months, 12–36 months, and >36 months). In other words, the development cohort was subjected to a three-classification condition. To determine reproducibility, twenty different numerical simulations were processed independently, and for each numerical simulation, 10-fold cross-validation was employed. Both the number of iterations from RBM and the stochastic gradient descent iterations were set as 100. Moreover, the mean of the simulation results indicated the classification accuracy for different combinations of the candidate variables. Through selection and comparison, according to the best classification accuracy, the final variables combination was settled.

#### Equation development using the T-S type fuzzy neural network

The final variables were used to establish a T-S type fuzzy neural network in the development cohort. The weighting function multiplier *W* was introduced for numerical indicators to establish a new haemodialysis initiation evaluation equation. After removing the outliers, 10-fold cross-validation was used for network training, and the parameters of the model were adjusted by particle swarm optimization (PSO)^28^. Based on a survival time of more or less than 36 months, patients in the development cohort were divided into two groups to determine the threshold and to evaluate the performance of the equation. Patients who survived for ≥36 months were assigned to the good survival group, whereas patients who survived for <36 months were assigned to the poor survival group. The sensitivity, specificity, and diagnostic accuracy of the 3-year mortality prediction after haemodialysis initiation were used to evaluate the equation’s performance. The candidate threshold with the best performance was determined as the final threshold. Meanwhile, we developed a logistic model with the same variables as the DIFE using the development cohort.

#### Equation validation

We divided the patients of the validation cohort into predicted timely and late start groups based on the DIFE threshold and plotted the Kaplan–Meier curves. Kaplan–Meier curves stratified by different eGFR thresholds (5, 6, 7, 8, and 9 mL/min/1.73 m^2^) were also created. The performance of the DIFE was validated based on the bootstrap-corrected Receiver-operator characteristic (ROC) area of the 3-year mortality prediction after haemodialysis initiation, and by comparing this with the logistic model and the conventional eGFR-based measurements, wherein eGFR was calculated by both the Chinese modified MDRD study (C-MDRD) equation and the Chronic Kidney Disease Epidemiology Collaboration (CKD-EPI) equation. Furthermore, we calculated the sensitivity, specificity, and diagnostic accuracy rate of the C-MDRD equation at various eGFR thresholds (5, 6, 7, 8, and 9 mL/min/1.73 m^2^) for 3-year mortality in the validation cohort.

### Statistical analysis

Continuous variables are expressed as the mean ± standard deviation or interquartile range. Categorical variables are expressed as frequencies (percentages). For the comparison of patient baseline data between the two cohort groups, continuous variables were evaluated using the Mann-Whitney U test, and categorical variables were evaluated using the χ^2^ test.

Statistical analysis was conducted using SPSS software (version 19.0; IBM, Armonk, NY, USA). All statistical tests were two-sided. Statistical significance was set at p < 0.05. All ANN models were constructed using MATLAB software (version 2011b; The MathWorks Inc., Natick, MA, USA), whereas R (version 3.4.1) open source software (R Foundation for Statistical Computing; www.Rproject.org) was used for validation.

## Results

### Cohort description

The baseline characteristics of the development cohort (n = 1281) and the validation cohort (n = 504) are presented in Table 1. Significant differences were observed in the body mass index (BMI), heart failure, vomiting, diabetes mellitus, haemoglobin level, blood urea nitrogen level, serum creatinine level, potassium level, calcium level, and phosphate level at the time of haemodialysis initiation between the two cohorts.

**Table 1:** Baseline characteristics of the development cohort and validation cohort.

	Development cohort (n = 1281)	Validation cohort (n = 504)	*P*
Sex (male, %)	59.9	62.5	0.307
Age (years)	54.0 ± 13.8	53.1 ± 15.1	0.221
Body mass index (kg/m^2^)	16.7 ± 23.5	16.8 ± 11.2	0.002
Symptoms at the beginning of haemodialysis (yes, %)			
Heart failure	29.1	41.3	<0.001
Vomiting	26.5	47.2	<0.001
Oedema (II° and above)	45.0	49.6	0.077
Uraemic encephalopathy	2.6	4.0	0.119
Diabetes (%)	22.4	29.2	0.003
Laboratory test levels at initiation			
Haemoglobin (g/dL)	8.5 ± 2.1	8.2 ± 2.1	0.033
Albumin (g/dL)	3.5 ± 0.7	3.5 ± 0.6	0.148
Blood urea nitrogen (mg/dL)	85.7 ± 5.7	91.5 ± 1.5	0.001
Serum creatinine (mg/dL)	10.1 ± 4.5	10.0 ± 4.2	0.778
Uraemia (µmol/L)	467.1 ± 163.1	449.7 ± 157.7	0.143
Potassium (mmol/L)	4.8 ± 0.9	4.9 ± 0.9	0.012
Sodium (mmol/L)	138.9 ± 4.3	139.1 ± 4.2	0.063
Calcium (mmol/L)	2.05 ± 0.32	1.97 ± 0.30	< 0.001
Phosphate (mmol/L)	2.0 ± 0.70	2.1 ± 0.70	0.028
eGFR (mL/min/1.73 m^2^)*	7.8 ± 3.9	7.6 ± 3.5	0.287
≤5 (%)	20.1	22.4	
5–10 (%)	61.1	60.7	
>10 (%)	18.8	16.9	
Died within 3 years of haemodialysis initiation (%)	12.2	11.3	0.969

Note:The conversion factor for the serum creatinine level in mg/dL to µmol/L is × 88.4; the conversion factor for the blood urea nitrogen level in mg/dL to mmol/L is × 0.357; the conversion factor for the haemoglobin and serum albumin levels in g/dL to g/L is ÷10.

Abbreviation: eGFR, estimated glomerular filtration rate.

*Calculated using the Chinese modified Modification of Diet in Renal Disease equation 7.

Within the first 3 years of haemodialysis, 156 patients died in the development cohort, and 57 patients died in the validation cohort. The 3-year mortality rate in the development cohort was 12.2 deaths per 100 patient-years, whereas the corresponding rate in the validation cohort was 11.3 deaths per 100 patient-years.

### Equation variable selection using the KLNN-RBM model

A total of 13 candidate variables were considered as the input variables in the KLNN-RBM model, including five variables (age, sex, serum creatinine level, blood urea nitrogen level, and serum albumin level) as part of the MDRD study equation 7^29^ and eight potential clinical factors (i.e., blood haemoglobin level, serum potassium level, serum phosphorus level, heart failure, vomiting, oedema grade 2+ and 3+, uraemia, encephalopathy, and diabetes mellitus). Classification accuracies obtained by the combination of different candidate variables are listed in Appendix Table 1. The best classification accuracy (64.30%) was achieved by using the following nine candidate variables: age, sex, serum creatinine level, serum albumin level, haemoglobin level, heart failure, diabetes mellitus, blood urea nitrogen level, and serum phosphorus level. All the aforementioned variables were retained in the DIFE.

### Equation development using a T-S type fuzzy neural network model

A T-S type fuzzy neural network model was established in the development cohort using the nine selected variables. *W* in the equation was a function of six numerical variables (i.e., serum creatinine level, age, serum albumin level, haemoglobin level, blood urea nitrogen level, and phosphorus level), and served as a multiplier representing the effects of these variables on the patients’ outcomes.

$$\begin{array}{lll}{\boldsymbol{Y}} & = & {P}_{15}+{P}_{1}\cdot W\cdot ({P}_{2}\cdot {{\rm{S}}{\rm{c}}{\rm{r}}}^{{P}_{3}}\cdot {({\rm{l}}{\rm{n}}({\rm{a}}{\rm{g}}{\rm{e}}))}^{{P}_{4}}\cdot {{\rm{A}}{\rm{l}}{\rm{b}}}^{{P}_{5}}\cdot {{\rm{H}}{\rm{b}}}^{{P}_{6}}\cdot {({\rm{l}}{\rm{n}}({\rm{B}}{\rm{U}}{\rm{N}}))}^{{P}_{7}}\cdot {{\rm{P}}}^{{P}_{8}}+{P}_{9}\cdot {e}^{{\rm{H}}{\rm{F}}}\\  &  & +\,{P}_{10}\cdot {e}^{{\rm{D}}{\rm{M}}}+{P}_{11}\cdot {e}^{{\rm{f}}{\rm{e}}{\rm{m}}{\rm{a}}{\rm{l}}{\rm{e}}}+{P}_{16})\\ W & = & (1+\exp ({T}_{1}+{T}_{2}{\rm{S}}{\rm{c}}{\rm{r}}+{T}_{3}{\rm{A}}{\rm{l}}{\rm{b}}+T4{\rm{H}}{\rm{b}}+{T}_{5}{\rm{l}}{\rm{n}}({\rm{B}}{\rm{U}}{\rm{N}})+{T}_{6}P){)}^{-1}\end{array}$$

The parameters of the equation variables are shown in Appendix Table 2.1, and the variable parameters of the multiplier are shown in Appendix Table 2.2. The candidate thresholds and the equation performance in the development cohort under every candidate threshold are shown in Appendix Table 3, and the threshold was considered to be 30. The logistic model was set up with the same nine variables, with a calibration value of 8.06 (*P* = 0.428) (shown in Table 2).

**Table 2:** Hazard Ratios for the Logistic Model in the Development Cohort.

Variables	β-coefficient	*P* value	Hazard Ratio (95% Confidence Interval)
Serum creatinine, per 1 mg/dL	0.022	0.451	1.023 (0.965, 1.083)
Age, per 1 y	0.016	0.022	1.016 (1.002, 1.030)
Serum albumin, per 1 mg/dL	−0.153	0.317	0.858 (0.653, 1.129)
Haemoglobin, per 1 g/dL	0.013	0.774	1.013 (0.927, 1.107)
Blood urea nitrogen, per 1 mg/dL	−0.007	0.046	0.993 (0.985, 1.000)
Phosphate, per 1 mmol/L	0.161	0.286	1.174 (0.874, 1.578)
Heart failure	0.183	0.364	1.201 (0.809, 1.783)
Diabetes	−0.104	0.620	0.902 (0.599, 1.358)
Male Sex	0.326	0.083	1.386 (0.958, 2.004)

Note: The conversion factor for the serum creatinine level in mg/dL to µmol/L is × 88.4; the conversion factor for the blood urea nitrogen level in mg/dL to mmol/L is × 0.357; the conversion factor for the haemoglobin and serum albumin levels in g/dL to g/L is ÷ 10.

### Performance of the equation in the validation cohort

We tested the accuracy of the equation in the validation cohort. The diagnostic accuracy rate of the equation was 72.42%, the specificity was 75.84%, and the sensitivity was 45.61%, with a threshold of 30, which remained the best among the different candidate thresholds. The 3-year mortality rates in the good survival group and poor survival group were 8.38 deaths per 100 patient-years and 19.40 deaths per 100 patient-years, respectively. The validation accuracies were similar to those in the development cohort and showed robust performance with the DIFE (listed in Table 3). Moreover, we evaluated the performance of the C-MDRD equation in the validation cohort based on the eGFR thresholds (5, 6, 7, 8, and 9 mL/min/1.73 m^2^). The best sensitivity, specificity, and diagnostic accuracy rates for the conventional assessment of 3-year mortality in the validation cohort were 19.3%, 77.0%, and 70.6% respectively, when the eGFR was 5 mL/min/1.73 m^2^; however, the values were all lower compared with those obtained using the DIFE (Table 3).

**Table 3:** Performance of the DIFE and the conventional eGFR-based assessment in the validation cohort

	Candidate thresholds	Poor quality of life group^a^ N	Good quality of life group^b^ N	Sensitivity %	Specificity %	Diagnostic accuracy rate %	Mortality rate^c^ in the poor quality of life group	Mortality rate^c^ in the good quality of life group
The DIFE	29.00	93	411	29.82	83.00	76.98	18.28	9.73
	30.00	113	370	45.61	75.84	72.42	19.40	8.38
	31.00	185	319	59.65	66.22	65.48	18.38	7.21
	32.00	239	265	77.19	56.38	58.73	18.41	4.91
	33.00	299	205	87.72	44.30	49.21	16.72	3.41
	**eGFR** ^**d**^ **thresholds**	**Early start group** ^**e**^ **N**	**Late start group** ^**f**^ **N**	**Sensitivity %**	**Specificity %**	**Diagnostic accuracy rate %**	**Mortality rate** ^**c**^ **in the early start group**	**Mortality rate** ^**c**^ **in the late start group**
The conventional eGFR-based assessment	5	391	113	19.3	77.0	70.6	11.8	9.7
	6	320	184	31.6	62.9	59.3	12.2	9.8
	7	252	252	42.1	49.0	48.2	13.1	9.5
	8	200	304	49.1	38.3	39.5	14.5	9.2
	9	134	370	66.7	25.7	30.4	14.2	10.2

Abbreviations: DIFE, dialysis initiation based on the fuzzy mathematics equation; eGFR, estimated glomerular filtration rate.

^a^Patients who survived <36 months were assigned to the poor survival group.

^b^Patients who survived ≥36 months were assigned to the good survival group.

^c^Mortality rate was reported as the rate per 100 patient-years.

^d^Calculated using the Chinese modified Modification of Diet in Renal Disease equation 7.

^e^Patients scheduled to undergo dialysis with eGFR greater than or equal to the threshold were assigned to the early start group.

^f^Patients scheduled to undergo dialysis with eGFR less than the threshold were assigned to the late start group.

### Comparison of the equation with the logistic model and the conventional eGFR-based assessments in the validation cohort

**Figure 3:**
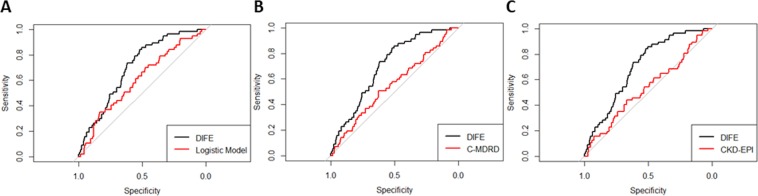
Receiver-operator characteristic (ROC) curves comparing the performances of the DIFE, the Logistic model and the estimated glomerular filtration rate (eGFR)-based conventional assessments in the validation cohort. (**A**) The DIFE compared with the Logistic Model. The area under ROC curve (AUC) for the DIFE equation was 0.70 (95% CI, 0.64–0.76), and the AUC for the Logistic Model was 0.60 (95% CI, 0.53–0.68). (**B**) The DIFE compared with the C-MDRD. The AUC for the C-MDRD equation was 0.55 (95% CI, 0.47–0.63). (**C**) The DIFE compared with the CKD-EPI. The AUC for the CKD-EPI was 0.53 (95% CI, 0.45–0.62). Abbreviations: DIFE, dialysis initiation based on the fuzzy mathematics equation; C-MDRD, Chinese modified Modification of Diet in Renal Disease equation; CKD-EPI, the Chronic Kidney Disease Epidemiology Collaboration equation.

**Figure 4:**
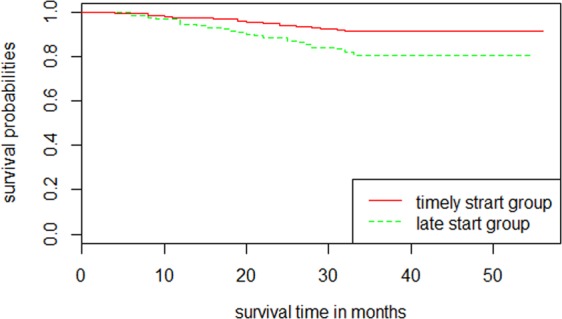
Kaplan*–*Meier survival curves of the patients in the validation cohort for initiating time to predictive death in 3 years separated by the DIFE threshold 30, *P* < 0.01 by log-rank test. Abbreviation: DIFE, dialysis initiation based on the fuzzy mathematics equation.

We found that the area under the receiver-operating characteristic curve (AUC) was 0.70 (95% confidence interval [CI], 0.64–0.76) for the DIFE and 0.60 (95% CI, 0.53–0.68) for the logistic model; the *P* value was 0.021 after 2000 times bootstrapping. When compared with the conventional eGFR-based assessments, the AUC was 0.55 for the C-MDRD study equation (95% CI, 0.47–0.63) and 0.53 for the CKD-EPI equation (95% CI, 0.45–0.62); the *P* values were 0.013 and 0.006 after 2000 times bootstrapping (Fig. 3). Furthermore, the Kaplan–Meier curves for the predictive timely and late start groups, based on the DIFE threshold, indicated a greater cumulative incidence of death in the predicted late start group (χ^2^_ = 212.1, *P < *0.001; Fig. 4). Moreover, there was no significant difference between the two groups, regardless of the eGFR threshold (*P* > 0.05; Appendix Fig. 1).

## Discussion

The GFR is commonly used to assess renal function and was also considered as a critical factor to evaluate the dialysis initiation time. However, eGFR alone is not sufficient to assess the dialysis initiation time^16,30,31^. Hence, we developed a novel equation, termed the DIFE, based on fuzzy mathematics and ANNs, which integrated multiple affecting factors, to assess dialysis initiation.

In this nation-wide prospective cohort, all the participants were enrolled from major haemodialysis centres in Mainland China. Nephrologists at these centres have considerable clinical experience and can make precise subjective judgments regarding dialysis initiation. Therefore, the new equation developed based on data from these centres, would ensure the optimum and precise assessment of dialysis start time in China.

Some observational studies and meta-analyses suggested that several clinical factors at dialysis initiation (e.g., haemoglobin value, serum phosphorus level, and heart failure) were associated with the prognosis of patients with ESRD, and some of these variables were already present in certain predictive models for CKD, renal failure, and risk of death^32–40^. Moreover, the KDOQI guidelines recommended that the decision to initiate dialysis should be based on an assessment of multiple clinical factors, including demographic factors, renal function, nutritional status, clinical signs and symptoms, and comorbidity^16^. Hence, we included these factors in the KLNN-RBM model as candidate variables, among which the signs and symptoms variable was the most frequently documented and routinely obtained.

The kernel logistic regression models and restricted Boltzmann machines are both proven techniques to handle feature selection and dimensionality reduction, and to determine the initial value of the model in classification situations. These two techniques have been used in disease diagnosis, gene screening, and other biological/ clinical studies^41–45^. However, to optimize the initial parameters and promote the classification capability of the logistic model, we combined a kernel logistic neural network with RBM and established the KLNN-RBM model. In our previous study, we validated KLNN-RBM in a single centre prospective cohort of dialysis patients. The results showed that the KLNN-RBM achieved higher accuracy compared with traditional logistic regression^27^. We also used six University of California Irvine (UCI) Machine Learning Repository datasets to test the performance of KLNN-RBM. The UCI Machine Learning Repository is a widely used primary source of machine learning data sets for the empirical analysis of machine learning algorithms, which has some biological and clinical datasets. The results also showed that the KLNN-RBM could achieve higher accuracy for the binary classification and multi-class classification problems^23^. These suggested that the KLNN-RBM model is an appropriate method for candidate variable screening.

In the present study, the results showed that the combination of sex, age, serum creatinine, blood urea nitrogen, serum albumin, haemoglobin, serum phosphorus, diabetes mellitus, and heart failure as equation variables resulted in the best accuracy. The clinical factors employed within the DIFE equation were consistent with those in the clinical guidelines and other cohort studies^10,16,34,35^. Based on these studies, we successfully determined the quantitative combination of these clinical factors for the first time. We initially compared the results of DIFE with a logistic model that had the same variables as the DIFE, and the DIFE demonstrated better model discrimination (the AUC of the DIFE was 0.70 *vs*. the AUC of the logistic model of 0.60). This result indicated that when integrating multiple variables, especially including some subjective judgments, fuzzy mathematics could be a more appropriate modelling method than traditional statistical methods. We also compared the AUC of the DIFE with those of two conventional eGFR-based assessments, and neither result was better than that of the DIFE (0.55 for the C-MDRD equation and 0.53 for the CKD-EPI equation). Therefore, the DIFE was more accurate to assess dialysis initiation than the conventional eGFR-based assessments. Furthermore, the Kaplan–Meier curves between the predictive timely and late start groups showed a significant difference based on the DIFE equation (*P < *0.001); however, the C-MDRD equation and the CKD-EPI equation did not show any difference in the start time of dialysis for any eGFR value (*P* > 0.05). This result indicated that the DIFE could be a more suitable assessment method for the timing of dialysis initiation compared with the eGFR based conventional assessment.

The following example shows the manner in which the DIFE can provide a quantified assessment of dialysis initiation. The decision for haemodialysis initiation was unclear in two ESRD patients with the same eGFR of 10 mL/min/1.73 m^2^ (clinical data shown in Table 4). If the other clinical factors were not considered, the eGFR value alone could not be used to make this decision. However, the DIFE equation yielded significantly different values: the value was 29.35 in patient A and 42.16 in patient B. Thus, the DIFE could directly identify that patient A required haemodialysis immediately, whereas patient B could undergo some preparation (e.g., vascular access placement) and undergo haemodialysis later. Liu reported that the major challenge for the management of patients with CKD in China is the lack nephrology specialists^46^. Patients with ESRD in China are usually treated by primary physicians who lack specialist training in nephrology and patients may receive inappropriate decisions on dialysis initiation. The Chinese government is implementing reforms in the medical education system to produce well-trained primary health-care providers; however, this will take time given the country’s vast need. The DIFE could help primary physicians to quantitative assess the initiation of dialysis through the DIFE value. Furthermore, to be applied conveniently, we developed a mobile phone application of the DIFE. The physician can get the DIFE value directly by entering the variables into a mobile phone.

**Table 4:** Assessment of two hypothetical patients with the same eGFR.

	Patient A, 65-year-old man with eGFR* 10 mL/min/1.73 m^2^	Patient B, 25-year-old man with eGFR* 10 mL/min/1.73 m^2^
Laboratory data		
BUN (mg/dL)	80.0	60
Scr (mg/dL)	6.6	9.0
Alb (g/dL)	3.0	4.0
Hb (g/dL)	8.0	8.0
P (mmol/L)	2.2	2.2
Clinical signs and symptoms		
Heart failure	No	No
Diabetes	Yes	No
DIFE value	29.35	42.16
Decision	Should start haemodialysis at once	Prepare and wait for haemodialysis

Note:The conversion factor for the serum creatinine level in mg/dL to µmol/L is × 88.4; the conversion factor for the blood urea nitrogen level in mg/dL to mmol/L is × 0.357; the conversion factor for the haemoglobin and serum albumin levels in g/dL to g/L is ÷ 10.

Abbreviation: eGFR, estimated glomerular filtration rate; BUN, blood urea nitrogen; Scr, serum creatinine; Alb, serum albumin; Hb, haemoglobin; P, serum phosphorus; DIFE, dialysis initiation based on the fuzzy mathematics equation.

*Calculated by the Chinese modified Modification of Diet in Renal Disease equation 7.

The present study had certain limitations. First, the DIFE included retrospective data from patients who had already undergone haemodialysis and may have excluded patients who died prior to haemodialysis initiation, which could lead to survivor bias. Second, because of some incomplete data in the retrospective cohorts, some clinical indicators, such as malnutrition symptoms and subjective global assessment (SGA), could not be included as candidate variables. We are conducting a prospective, multicentre, randomized, controlled trial concerned nutrition status, including SGA and malnutrition symptoms, to verify and improve the equation (the clinicalTrials.gov ID is NCT 03385902). Although neural network techniques have been developed in a wide range of applications in recent years, and are proven to be superior to conventional statistical models to assess initiation of dialysis, how to optimize the structure and parameters in neural networks remains a challenge.

In the present study, we developed a novel fuzzy neural network model to evaluate the optimal time of haemodialysis initiation in a Chinese ESRD population. The variables in the equation included the clinical indicators of haemodialysis initiation, and the performance of the equation was found to be more precise than conventional eGFR-based assessments. This equation may be a helpful tool to quantitatively evaluating the initiation of haemodialysis.

## Supplementary information

appendix tables and figures

## Data Availability

The datasets generated during the current study are available from the corresponding author on reasonable request.

## References

[1] Saggi SJ (2012). Considerations in the optimal preparation of patients for dialysis. Nat Rev Nephrol.

[2] Abra G, Kurella Tamura M (2012). Timing of initiation of dialysis: time for a new direction?. Curr Opin Nephrol Hypertens.

[3] Leurs P, Machowska A, Lindholm B (2015). Timing of dialysis initiation: when to start? Which treatment?. J Ren Nutr.

[4] Rivara MB, Mehrotra R (2017). Timing of Dialysis Initiation: What Has Changed Since IDEAL?. Seminars in nephrology.

[5] Hakim RM, Lazarus JM (1995). Initiation of dialysis. J Am Soc Nephrol.

[6] Churchill DN (1997). An evidence-based approach to earlier initiation of dialysis. Am J Kidney Dis.

[7] Jansen MA (2001). Renal function and nutritional status at the start of chronic dialysis treatment. J Am Soc Nephrol.

[8] U.S. Renal Data System, USRDS 2009 Annual Data Report: Atlas of Chronic Kidney Disease and End-Stage Renal Disease in the United States. (National Institutes of Health, National Institute of Diabetes and Digestive and Kidney Diseases, Bethesda, MD, 2009).

[9] Rosansky SJ, Eggers P, Jackson K, Glassock R, Clark WF (2011). Early start of hemodialysis may be harmful. Arch Intern Med.

[10] Hwang SJ (2010). Impact of the clinical conditions at dialysis initiation on mortality in incident haemodialysis patients: a national cohort study in Taiwan. Nephrol Dial Transplant.

[11] Wong MG (2014). Association between GFR estimated by multiple methods at dialysis commencement and patient survival. Clin J Am Soc Nephrol.

[12] Crews DC (2014). Comparative effectiveness of early versus conventional timing of dialysis initiation in advanced CKD. Am J Kidney Dis.

[13] Scialla JJ (2014). An instrumental variable approach finds no associated harm or benefit with early dialysis initiation in the United States. Kidney Int.

[14] Cooper BA (2010). A randomized, controlled trial of early versus late initiation of dialysis. N Engl J Med.

[15] Berns JS (2015). Clinical Decision Making in a Patient with Stage 5 CKD–Is eGFR Good Enough?. Clin J Am Soc Nephrol.

[16] National Kidney, F. KDOQI Clinical Practice Guideline for Hemodialysis Adequacy: 2015 update. *Am J Kidney Dis* **66**, 884–930, 10.1053/j.ajkd.2015.07.015 (2015). 10.1053/j.ajkd.2015.07.015 26498416

[17] Rivara MB, Mehrotra R (2014). Is early initiation of dialysis harmful?. Semin Dial.

[18] Zadeh LA (1965). Fuzzy sets*. Information & Control.

[19] Terano, T., Asai, K. & Sugeno, M. *Fuzzy systems theory and its applications*. (Academic Press Professional, Inc., 1992).

[20] Tangri N, Ansell D, Naimark D (2008). Predicting technique survival in peritoneal dialysis patients: comparing artificial neural networks and logistic regression. Nephrol Dial Transplant.

[21] Tangri N, Ansell D, Naimark D (2011). Determining factors that predict technique survival on peritoneal dialysis: application of regression and artificial neural network methods. Nephron Clin Pract.

[22] Marshall MR, Song Q, Ma TM, MacDonell SG, Kasabov NK (2005). Evolving connectionist system versus algebraic formulas for prediction of renal function from serum creatinine. Kidney Int.

[23] Lv, Q. *et al*. A kernel logistic neural network based on restricted Boltzmann machine. *In International Conference on Informative and Cybernetics for Computational Social Systems* 1–6 (2016).

[24] Takagi T, Sugeno M (1993). Fuzzy identification of systems and its applications to modeling and control. Readings in Fuzzy Sets for Intelligent Systems.

[25] Du G, Jiang Z, Diao X, Yao Y (2013). Intelligent ensemble T-S fuzzy neural networks with RCDPSO_DM optimization for effective handling of complex clinical pathway variances. Comput Biol Med.

[26] Du G, Jiang Z, Diao X, Ye Y, Yao Y (2012). Variances handling method of clinical pathways based on T-S fuzzy neural networks with novel hybrid learning algorithm. J Med Syst.

[27] Lv, Q. *et al*. *In International Conference on Informative and Cybernetics for Computational Social Systems*. 1–6.

[28] Kennedy, J. & Eberhart, R. *Particle swarm optimization*. (Springer US, 2011).

[29] Levey AS (1999). A more accurate method to estimate glomerular filtration rate from serum creatinine: a new prediction equation. Modification of Diet in Renal Disease Study Group. Ann Intern Med.

[30] Eloot S (2011). Estimated glomerular filtration rate is a poor predictor of concentration for a broad range of uremic toxins. Clin J Am Soc Nephrol.

[31] Grootendorst DC (2011). The MDRD formula does not reflect GFR in ESRD patients. Nephrol Dial Transplant.

[32] Kataoka H (2015). Relationship between anaemia management at haemodialysis initiation and patient prognosis. Nephrology (Carlton).

[33] Lu YA (2015). Serum phosphate as an additional marker for initiating hemodialysis in patients with advanced chronic kidney disease. Biomed J.

[34] Kaizu, K. *et al*. Clinical profiles and outcomes of end-stage renal failure patients with late initiation of renal replacement therapy based on uremic symptoms under intensive renoprotective therapies. *Am J Nephrol* **22**, 521–531, doi:65290 (2002). 10.1159/000065290 12381954

[35] Tangri N (2016). Multinational Assessment of Accuracy of Equations for Predicting Risk of Kidney Failure: A Meta-analysis. JAMA.

[36] Tangri, N. *et al*. A Dynamic Predictive Model for Progression of CKD. *Am J Kidney Dis*, 10.1053/j.ajkd.2016.07.030 (2016). 10.1053/j.ajkd.2016.07.030 27693260

[37] Tangri N (2011). A predictive model for progression of chronic kidney disease to kidney failure. JAMA.

[38] van de Luijtgaarden MW (2012). Factors influencing the decision to start renal replacement therapy: results of a survey among European nephrologists. Am J Kidney Dis.

[39] Lassalle M (2010). Age and comorbidity may explain the paradoxical association of an early dialysis start with poor survival. Kidney Int.

[40] Rivara MB (2017). Indication for Dialysis Initiation and Mortality in Patients With Chronic Kidney Failure: A Retrospective Cohort Study. Am J Kidney Dis.

[41] Zhang, J. *et al*. Machine-learning algorithms define pathogen-specific local immune fingerprints in peritoneal dialysis patients with bacterial infections. *Kidney Int*, 10.1016/j.kint.2017.01.017 (2017). 10.1016/j.kint.2017.01.017 PMC5484022 28318629

[42] Yoneoka D, Saito E, Nakaoka S (2016). New algorithm for constructing area-based index with geographical heterogeneities and variable selection: An application to gastric cancer screening. Sci Rep.

[43] Wei P, Tang H, Li D (2012). Insights into pancreatic cancer etiology from pathway analysis of genome-wide association study data. PLoS One.

[44] Fong Y, Datta S, Georgiev IS, Kwong PD, Tomaras GD (2015). Kernel-based logistic regression model for protein sequence without vectorialization. Biostatistics.

[45] Hinton GE, Osindero S, Teh YW (2006). A fast learning algorithm for deep belief nets. Neural Comput.

[46] Liu ZH (2013). Nephrology in china. Nat Rev Nephrol.

