# Supplementary material for: An Equation Based on Fuzzy Mathematics to Assess the Timing of Haemodialysis Initiation
Source: Sci Rep. 2019 Apr 10;9:5871. doi: 10.1038/s41598-018-37762-6 (PMC6458145; doi:10.1038/s41598-018-37762-6)
Supplement: Supplementary file 1 — appendix tables and figures [file 41598_2018_37762_MOESM1_ESM.pdf]

An Equation Based on Fuzzy Mathematics to Assess the Timing of Haemodialysis Initiation

- A Cohort Study

Ying Liu, MD[1,2,3], Degang Wang, PhD[4], Xiangmei Chen, MD, PhD[5], Xuefeng Sun, MD, PhD[5], Wenyan Song, PhD[6], Hongli Jiang, MD[7], Wei Shi, MD, PhD[8], Wenhu Liu, MD, PhD[9], Ping Fu, MD, PhD[10], Xiaoqiang Ding, MD, PhD[11], Ming Chang, MM[12], Xueqing Yu, MD, PhD[13], Ning Cao, PhD[14], Menghua Chen, PhD[15], Zhaohui Ni, MD, PhD[16], Jing Cheng, MD, PhD[17], Shiren Sun, MD, PhD[18], Huimin Wang, MM[19],Yunyan Wang, BSM[20], Bihu Gao, PhD[21], Jianqin Wang, PhD[22], Lirong Hao, MD, PhD[23], Suhua Li, MD[24], Qiang He, PhD[25], Hongmei Liu, BSM[26], Fengmin Shao, PhD[27], Wei Li, PhD[28], Yang Wang, MPH[28], Lynda Szczech, MD, MSCE[29], Qiuxia Lv, ME[4], Xianfeng Han, MM[1,2], Luping Wang, MM[1,2], Ming Fang, MD[1,2,3], Zach Odeh, MS[1,2], Ximing Sun, PhD[4], Hongli Lin, MD, PhD[1,2,3*]

[1]Dalian Medical University Graduate School, Dalian, China

[2]Department of Nephrology, The First Affiliated Hospital of Dalian Medical University, Liaoning Province Translational Medicine Research Center of Kidney Disease, Dalian, China

[3]Kidney Research Institute of Dalian Medical University, Dalian, China

[4]School of Control Science and Engineering, Dalian University of Technology, Dalian, China

[5]Department of Nephrology, Chinese PLA General Hospital, Chinese PLA Institute of Nephrology, State Key Laboratory of Kidney Diseases, National Clinical Research Center for Kidney Diseases, Beijing Key Laboratory of Kidney Disease Research, Beijing, China

[6]School of Economics, Dongbei University of Finance and Economics, Dalian, China

[7]Blood Purification Center, The First Affiliated Hospital of Xi'an Jiaotong University, Xi'an, China

[8]Division of Nephrology, Guangdong General Hospital, Guangdong Academy of Medical Sciences, Guangzhou, China

[9]Division of Nephrology, Beijing Friendship Hospital, Capital Medical University, Beijing, China
[10]Kidney Research Institute, Division of Nephrology, West China Hospital of Sichuan University, Chengdu, China
[11]Division of Nephrology, Zhongshan Hospital, Fudan University, Shanghai, China
[12]Division of Nephrology, Dalian Municipal Central Hospital, Dalian, China
[13]Department of Nephrology, The First Affiliated Hospital, Sun Yat-sen University, Key Laboratory of Nephrology, Ministry of Health of China, Guangzhou, China
[14]Blood Purification Center, General Hospital of Shenyang Military Area Command, Shenyang, China
[15]Department of Nephrology, General Hospital of Ningxia Medical University, Yinchuan, China
[16]Department of Nephrology, Renji Hospital, School of Medicine, Shanghai Jiaotong University, Shanghai, China
[17]Division of Nephrology, Huashan Hospital, Fudan University, Shanghai, China
[18]Department of Nephrology, Xijing Hospital, the Fourth Military Medical University, Xi'an, China
[19] Division of Nephrology，General Hospital of Benxi Iron and Steel Co., Ltd., Benxi, China
[20]Blood Purification Center, Daping Hospital & Surgery Institute, the Third Military Medical University, Chongqing, China
[21]Division of Nephrology，Affiliated Zhongshan Hospital of Dalian University, Dalian, China
[22]Division of Nephrology, Lanzhou University Second Hospital, Lanzhou, China
[23]Division of Nephrology, the First Affiliated Hospital of Harbin Medical University, Harbin, China
[24]Division of Nephrology, the First Affiliated Hospital of Xinjiang Medical University, Urumchi, China
[25]Division of Nephrology, Zhejiang Provincial People's Hospital, Hangzhou, China
[26]Division of Nephrology, An Steel Group Hospital, Anshan, China

[27]Blood Purification Center, The People's Hospital of Zhengzhou University & Henan Provincial People's Hospital, Zhengzhou, China
[28]Medical Research & Biometrics Center, Fuwai Hospital, Chinese Academy of Medical Sciences, Beijing, China
[29]FibroGen, Inc., San Francisco, CA, USA.

Correspondence: Dr. Hongli Lin, Department of Nephrology, the First Affiliated Hospital of Dalian Medical University, No. 222, Zhongshan Road, Dalian 116011, China. E-mail: hllin@dlmedu.edu.cn

Appendix Table 1. Classification accuracies of different combinations of candidate variables based on the KLNN-RBM

Combinations of candidate variables	Classification accuracy (%)
Scr, BUN, age, sex, Alb	58.83
Scr, BUN, age, sex, Alb, Hb	62.52
Scr, BUN, age, sex, Alb, HF	60.43
Scr, BUN, age, sex, Alb, P	60.18
Scr, BUN, age, sex, Alb, DM	61.02
Scr, BUN, age, sex, Alb, Hb, HF	61.08
Scr, BUN, age, sex, Alb, Hb, P	60.63
Scr, BUN, age, sex, Alb, Hb, HF, DM	62.75
Scr, BUN, age, sex, Alb, Hb, P, HF, DM	64.30
Scr, BUN, age, sex, Alb, Hb, K, P, HF, DM	62.72
Scr, BUN, age, sex, Alb, Hb, P, HF, DM, vomiting	61.72
Scr, BUN, age, sex, Alb, Hb, P, HF, DM, vomiting, oedema	62.77
Scr, BUN, age, sex, Alb, Hb, K, P, HF, DM, UE	63.05
Scr, BUN, age, sex, Alb, Hb, P, HF, DM, vomiting, oedema, UE	63.12
Scr, BUN, age, sex, Alb, Hb, K, P, HF, DM, vomiting, oedema, UE	63.28

Abbreviations: KLNN-RBM, kernel logistic neural network–restricted Boltzmann machine; Scr, serum creatinine; BUN, blood urea nitrogen; Alb, serum albumin; Hb, haemoglobin; K, serum potassium; P, serum phosphorus; HF, heart failure; DM, diabetes; UE, uremic encephalopathy.

Appendix Table 2.1 Parameters of the DIFE equation

**Parameters**	P_1	P_2	P_3	P_4	P_5	P_6	P_7	P_8	P_9	P_{10}	P_{11}	P_{12}	P_{13}	P_{14}	P_{15}	P_{16}
**Values**	79.5909	3.7110	−0.0163	−0.1652	0.0113	0.0056	−0.0318	−0.0021	−0.7073	−0.7191	0.6658	0.0340	0.0412	0.0268	−132.0238	0.0561

Abbreviations: DIFE, dialysis initiation based on the fuzzy mathematics equation.

Appendix Table 2.2 Parameters of the multiplier *W*

**Parameters**	T_1	T_2	T_3	T_4	T_5	T_6
**Values**	−0.8755	0.0190	−1.4527	−0.0523	0.0196	0.0966

Appendix Table 3. Performance of the DIFE for the development cohort

Candidate thresholds	Poor quality of life group[a] N	Good quality of life group[b] N	Sensitivity %	Specificity %	Diagnostic accuracy rate %	Mortality Rate in the poor quality of life group	Mortality rate in the good quality of life group
29.00	184	1097	17.95	86.13	77.83	15.22	11.67
30.00	279	1002	25.64	78.76	72.29	14.34	11.58
31.00	412	869	33.97	68.09	63.93	12.86	11.85
32.00	545	736	42.95	57.51	55.74	12.29	12.09
33.00	676	605	54.49	47.47	48.32	12.57	11.74

Abbreviations: DIFE, dialysis initiation based on the fuzzy mathematics equation.

[a]Patients who survived < 36 months were assigned to the poor survival group.

[b]Patients who survived ≥ 36 months were assigned to the good survival group.

[c] Mortality rate was reported as the rate per 100 patient-years.

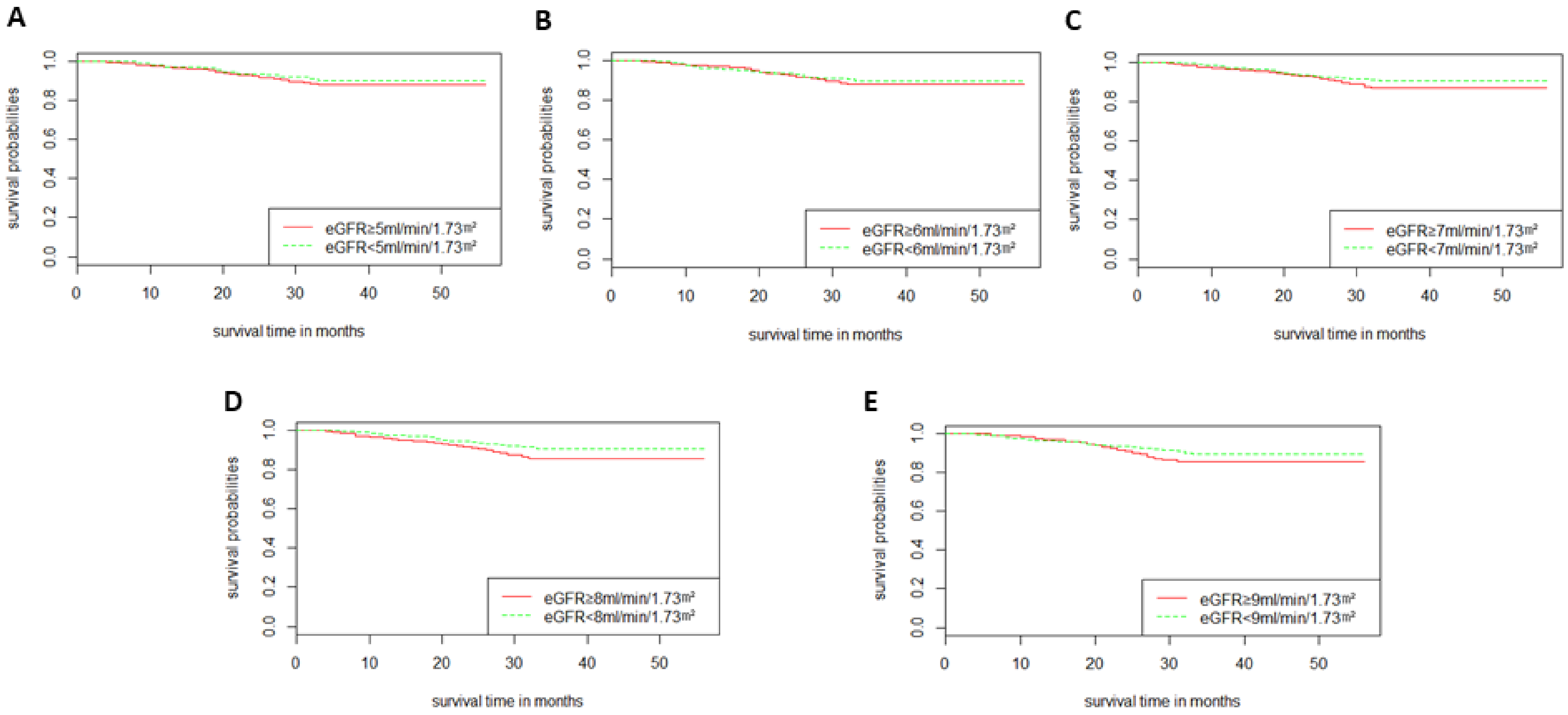

Appendix Figure 1. Kaplan–Meier survival curves of the patients in the validation cohort for initiating time to predictive death in 3 years separated by the different eGFR thresholds. A. eGFR threshold was 5 mL/min/1.73m2, P = 0.544 by the log-rank test. B. eGFR threshold was 6 mL/min/1.73m2, P = 0.609 by the log-rank test. C. eGFR threshold was 7 mL/min/1.73m2, P = 0.223 by the log-rank test. D. eGFR threshold was 8 mL/min/1.73m2, P = 0.065 by the log-rank test. E. eGFR threshold was 9 mL/min/1.73m2, P = 0.225 by the log-rank test. eGFR was calculated using the Chinese modified Modification of Diet in Renal Disease equation. Abbreviation: eGFR, estimated glomerular filtration rate.
